# Towards unlocking the biocontrol potential of *Pichia kudriavzevii* for plant fungal diseases*: *in vitro and in vivo assessments with candidate secreted protein prediction

**DOI:** 10.1186/s12866-023-03047-w

**Published:** 2023-11-18

**Authors:** Bassma Mahmoud Elkhairy, Nabil Mohamed Salama, Abdalrahman Mohammad Desouki, Ashraf Bakry Abdelrazek, Khaled Abdelaziz Soliman, Samir Abdelaziz Ibrahim, Hala Badr Khalil

**Affiliations:** 1 Department of Genetics, Faculty of Agriculture, Ain Shams University, 68 Hadayek Shoubra, Cairo, 11241 Egypt; 2 Biotechnology Labs, NanoFab Technology Company, 6th October, Giza, Egypt; 3 Department of Plant Pathology, Faculty of Agriculture, Ain Shams University, Postal Code, 68 Hadayek Shoubra, Cairo, 11241 Egypt; 4 Biological Sciences Department, College of Science, King Faisal University, Hofuf, Kingdom of Saudi Arabia

**Keywords:** Pichia kudriavzevii, Plant pathogenic fungi, Biocontrol agent, Secreted proteins, Antifungal inhibitors, Hydrolase proteins

## Abstract

**Background:**

Plant fungal pathogens cause substantial economic losses through crop yield reduction and post-harvest storage losses. The utilization of biocontrol agents presents a sustainable strategy to manage plant diseases, reducing the reliance on hazardous chemical. Recently, *Pichia kudriavzevii* has emerged as a promising biocontrol agent because of its capacity to inhibit fungal growth, offering a potential solution for plant disease management.

**Results:**

Two novel *Pichia kudriavzevii* strains, *Pk_*EgyACGEB_O1 and *Pk_*EgyACGEB_O2, were isolated from olive brine samples. The microscopic characterization of the strains revealed similar structures. However, there were noticeable differences in their visual morphology. Based on their internal transcribed spacer (ITS) DNA sequences, *Pk_*EgyACGEB_O1 and *Pk_*EgyACGEB_O2 strains assigned by GenBank IDs MZ507552.1 and MZ507554.1 shared high sequence similarity (~ 99.8% and 99.5%) with *P. kudriavzevii*, respectively. Both strains were evaluated in vitro against plant pathogenic fungi. The strains revealed the ability to consistently inhibit fungal growth, with *Pk_*EgyACGEB_O2 showing higher effectiveness. In addition, both *P. kudriavzevii* strains effectively controlled grey mold disease caused by *B. cinerea* in golden delicious apples, suggesting their potential as sustainable and eco-friendly biocontrol agents for post-harvest diseases. Based on a comprehensive bioinformatics pipeline, candidate-secreted proteins responsible for the potent antifungal activity of *P. kudriavzevii* were identified. A total of 59 proteins were identified as common among the *P. kudriavzevii* CBS573, SD108, and SD129 strains. Approximately 23% of the secreted proteins in the *P. kudriavzevii* predicted secretome are hydrolases with various activities, including proteases, lipases, glycosidases, phosphatases, esterases, carboxypeptidases, or peptidases. In addition, a set of cell-wall-related proteins was identified, which might enhance the biocontrol activity of *P. kudriavzevii* by preserving the structure and integrity of the cell wall. A papain inhibitor was also identified and could potentially offer a supplementary defense against plant pathogens.

**Conclusion:**

Our results revealed the biocontrol capabilities of *P. kudriavzevii* against plant pathogenic fungi. The research focused on screening novel strains for their ability to inhibit the growth of common pathogens, both in vitro and in vivo. This study shed light on how *P. kudriavzevii* interacts with fungal pathogens. The findings can help develop effective strategies for managing plant diseases.

**Supplementary Information:**

The online version contains supplementary material available at 10.1186/s12866-023-03047-w.

## Background

Fungal diseases are a huge threat to all plants and their products. These diseases can cause various symptoms in plants, from leaf spots to wilting and rotting, leading to reduced productivity, yield, and quality [1]. Fungal pathogens can also infect post-harvest plant products during storage and transportation, resulting in spoilage, loss of quality, and health risks [2]. Fungal diseases are often difficult to control, as fungi can survive in the soil and on plant debris for extended periods. They can also spread rapidly through wind, water, and insects [3]. Fungal spoilage causes degradation of the sensory attributes of the plant product, such as appearance, texture, flavour, and aroma. The decay can also cause the production of mycotoxins, which are toxic metabolites produced by some fungi and can pose a serious risk to human and animal health if ingested [4].

Efficient management strategies are required to minimise the impact of fungi on global food security. Farmers and plant breeders can use various methods to manage fungal infections, including cultural practices, chemical control, and biological control [5]. Cultural practices such as crop rotation, soil sterilisation, and the removal of diseased plant material can help to reduce the spread of fungal pathogens [6]. Chemical control involves using fungicides, which can be effective but can also negatively impact the environment and human health [7]. Chemical control uses fungicides that inhibit fungal growth, reproduction, or survival. Although chemical control methods have proven successful in the management of fungal diseases, they still pose certain challenges [8]. One major challenge is the development of resistance in fungal pathogens to commonly used fungicides, leading to reduced efficacy and increased use of chemicals. Moreover, chemical control methods can have negative impacts on the environment and human health, such as the contamination of soil and water resources and the potential for toxic residues in food products [9]. To tackle these issues, it is imperative to conduct research aimed at devising alternative and environmentally friendly approaches to managing fungal infections. In addition, there is a need for further research into the mechanisms of fungal pathogenesis and the genetic basis of plant resistance to fungal diseases, which can inform the development of more effective management strategies.

In recent years, there has been growing interest in using biological control as a potential alternative approach for managing fungal diseases in plants. Biological control involves the use of natural enemies, such as beneficial microorganisms, to control the growth and spread of fungal pathogens [10]. Biological control agents can either be used preventively to reduce the risk of pathogen establishment or as curative measures to control established infections. The potential benefits of using biological control for managing fungal diseases in plants are numerous. Unlike chemical control methods, biological control is generally considered to be safer for the environment and human health. It also offers a sustainable approach to disease management, as biological control agents can be naturally occurring or introduced and established in the environment. Furthermore, biological control can reduce the development of resistance in fungal pathogens as the use of natural enemies exerts selective pressure on the pathogen population [11].

Despite these potential benefits, there are also challenges associated with the use of biological control for managing fungal diseases in plants. One major challenge is the variability in the effectiveness of biological control agents against different fungal pathogens and under various environmental conditions [12]. In addition, the regulatory approval process for biological control agents can be time-consuming and expensive. Therefore, there is a need to develop effective and practical methods for delivering biological control agents to plants to maximise their effectiveness [10].

The *Pichia* genus is a group of yeast-like fungi that are classified under the family Saccharomycetaceae and comprise approximately 100 different species [13]. They are found in a wide range of environments, including soil, water, plants, and animals. In addition to their industrial importance, several species of *Pichia* also have ecological roles in the environment. They can be found as plant endophytes, meaning they live inside the plant, causing no harm, where they can promote plant growth and provide protection against pathogenic fungi [14]. The potential of many species of *Pichia* as biocontrol agents for fungal diseases in plants has been recently investigated. These species have been shown to have antifungal properties, producing compounds that inhibit the growth of pathogenic fungi [15]. They can also induce plant defence responses, making the plant more resistant to fungal infections [16]. Despite the potential of many species of *Pichia* as biocontrol agents, there are challenges associated with their implementation. One challenge is the variability in effectiveness against different fungal pathogens under a wide range of environmental conditions. Additionally, there is a need for further research to understand the mechanisms of *Pichia*-induced plant defence responses and optimise the delivery of *Pichia* to plants. However, there are also opportunities for the use of species of *Pichia* as biocontrol agents. They offer a sustainable and environmentally friendly alternative to chemical fungicides and can potentially reduce the development of resistance to fungal pathogens [17].

Several species of *Pichia* have gained attention as biocontrol agents due to their ability to inhibit the growth of various plant pathogens. The studies suggest that species of *Pichia,* including *P. anomala*, *P. guilliermondii*, *P. kluyveri*, *P. membranifaciens*, and *P. pastoris,* have the potential to be effective biocontrol agents against a range of plant pathogens [14–16]. They produce antifungal compounds that inhibit the growth of plant pathogens and have been shown to control post-harvest diseases in fruits and vegetables. One particular species that is promising as a potential biocontrol agent is *P. anomala* [15]. This is known for its ability to produce the antifungal compound phenylacetic acid, which inhibits the growth of several plant pathogenic fungi. *P. anomala* has also been found to induce the expression of plant defence-related genes, resulting in increased resistance to fungal infections [18]. *P. guilliermondii* has been studied as a biocontrol agent against various plant pathogens, including *Fusarium oxysporum*, *Alternaria alternata*, and *Rhizoctonia solani*. Studies have shown that *P. guilliermondii* can produce antifungal compounds that inhibit the growth of these pathogens [19]. *P. kluyveri* has been investigated for its ability to control post-harvest diseases in fruits and vegetables as it can inhibit the growth of various plant pathogens, including *Botrytis cinerea* and *Penicillium expansum* [20]. *P. kluyveri* has also been found to produce antifungal compounds that inhibit the growth of these pathogens [21]. Further studies are also required to determine their efficacy in different crop systems and under different environmental conditions.

Using *P. kudriavzevii* as a biocontrol agent may offer a sustainable and effective alternative to chemical fungicides in agriculture. However, further research is needed to determine the most effective application strategies and formulations for its use in different crop systems and under different environmental conditions. Studies have shown that *P. kudriavzevii* can inhibit the growth of various plant pathogens in various crops. One study demonstrated the application of *P. kudriavzevii* to retard fungal decay by influencing the fungal community during cherry tomato fruit storage [22]. In another study, *P. kudriavzevii* was shown to produce volatile organic compounds that inhibited the growth of *B. cinerea*, thereby reducing gray mold disease in grapevines [23]. Recently, a study examined the effects of calcium ascorbate on the antioxidant capacity and physiological activity of *P. kudriavzevii*. Furthermore, Ca^+2^ exhibited a synergistic promotion effect, which enhanced the biocontrol efficacy [24].

This study aims to identify and assess the potential of using more potent strains of *P. kudriavzevii* as biocontrol agents for controlling plant fungal pathogens both in vitro and in vivo. The investigation focuses on screening novel strains of *P. kudriavzevii* for their ability to inhibit the growth of common plant fungal pathogens, including those that cause diseases, such as *Aspergillus terreus, Botrytis cinerea, Bipolaris sp., Cochliobolus spicifer, Rhizoctonia solani, Fusarium solani, Fusarium circinatum,* and *Fusarium spp*. We also aim to advance our understanding of the art of science behind the interaction between *P. kudriavzevii* and fungal plant pathogens, which could lead to developing more sustainable and effective approaches for disease management in agriculture.

## Results

### Morphological and microscopic characterization of isolated *Pichia*-like colonies

During the fermentation stage of two olive brine samples, *Pichia*-like colonies were isolated and examined. Previous studies demonstrated the suitability of olive brine as a valuable source for isolating different species of Pichia, including *P. membranifaciens, P. anomala,* and *P. kluyveri* [25–28]. Building upon this finding, we have chosen to utilise olive brine as our primary resource for sample collection. Here, we aimed to capture the potential diversity of *Pichia* species that thrive in this specific environment. As these colonies underwent aerobic growth, they expanded in size and displayed various features such as tan-white colour, dull texture, and smoothness as described by Kreger-van Rij [29]. Some colonies even had a powdery and chalky consistency.

**Fig. 1:**
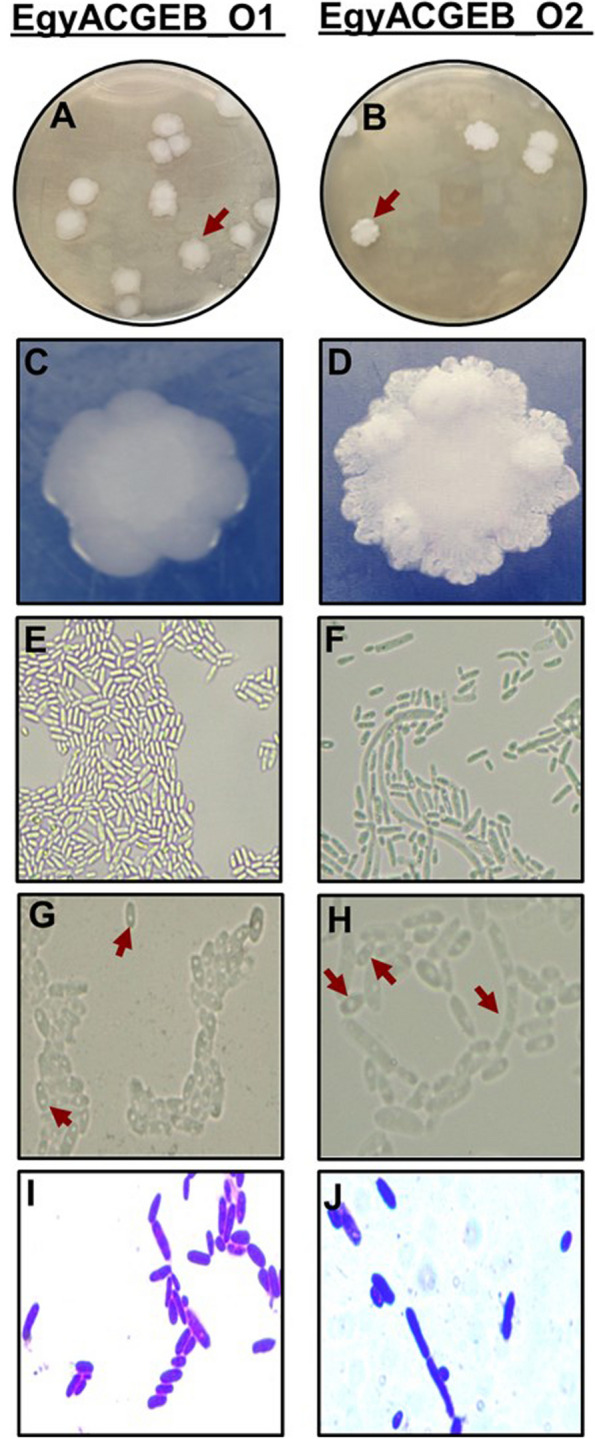
Morphological and microscopic characterization of EgyACGEB_O1 and EgyACGEB_O2 isolates collected from olive brine fermented samples. EgyACGEB_O1 and EgyACGEB_O2 isolates are grown on YMPG agar medium at 25° C for three day. **A-B** Single colonies of the two olive brine isolates grown on YMPG agar medium reveal *Pichia*-like creamy color colonies, **C** texture of butyrous, and **D** chalky dry climbing pellicle formation. **E–F** The microscopic appearance of unstained EgyACGEB_O1 and EgyACGEB_O2 isolates under 40X magnification, scale bar = 50 µm, **G-H** and under 100X magnification, scale bar = 20 µm reveals sopheric, ascospore, and pseudohyphae formations (red arrows). **I-J** Crystal violet stained cells under 100X magnification, scale bar = 20 µm

Further analysis was conducted by growing selected single colonies on yeast extract malt extract peptone glucose (YMPG) agar medium to better understand their characteristics. Upon observation, the colonies appeared as creamy-coloured structures reminiscent of *Pichia*. Two different colonies were isolated based on their shape, size, and texture. The first colony, named EgyACGEB_O1, exhibited a butyrous texture (Fig. 1A, C). The creamy-coloured appearance of the colony, coupled with its butyrous texture, made it stand out from the other colonies. The second colony, named EgyACGEB_O2, was distinctly different, as it appeared chalky and dry, with a climbing pellicle forming on its surface (Fig. 1B, D).

The EgyACGEB_O1 and EgyACGEB_O2 *Pichia*-like isolates were examined under the light microscope at both 40X and 100X magnifications to closely capture their characteristics (Fig. 1E-H). The cells were observed to have spheric, ascospore, and pseudohyphae formations, which were highlighted by red arrows in (Fig. 1G, H). These formations are indicative of the structure and growth patterns of the cells. In addition, crystal violet staining was used to provide more contrast and allow for clearer observation of the cells (Fig. 1I, J). The staining revealed more details about the structures present in the cells and helped in identifying them. This microscopic characterization of *Pichia* provided information about the morphology, growth pattern, and cellular structure.

### Molecular identification of EgyACGEB_O1 and EgyACGEB_O2

**Fig. 2:**
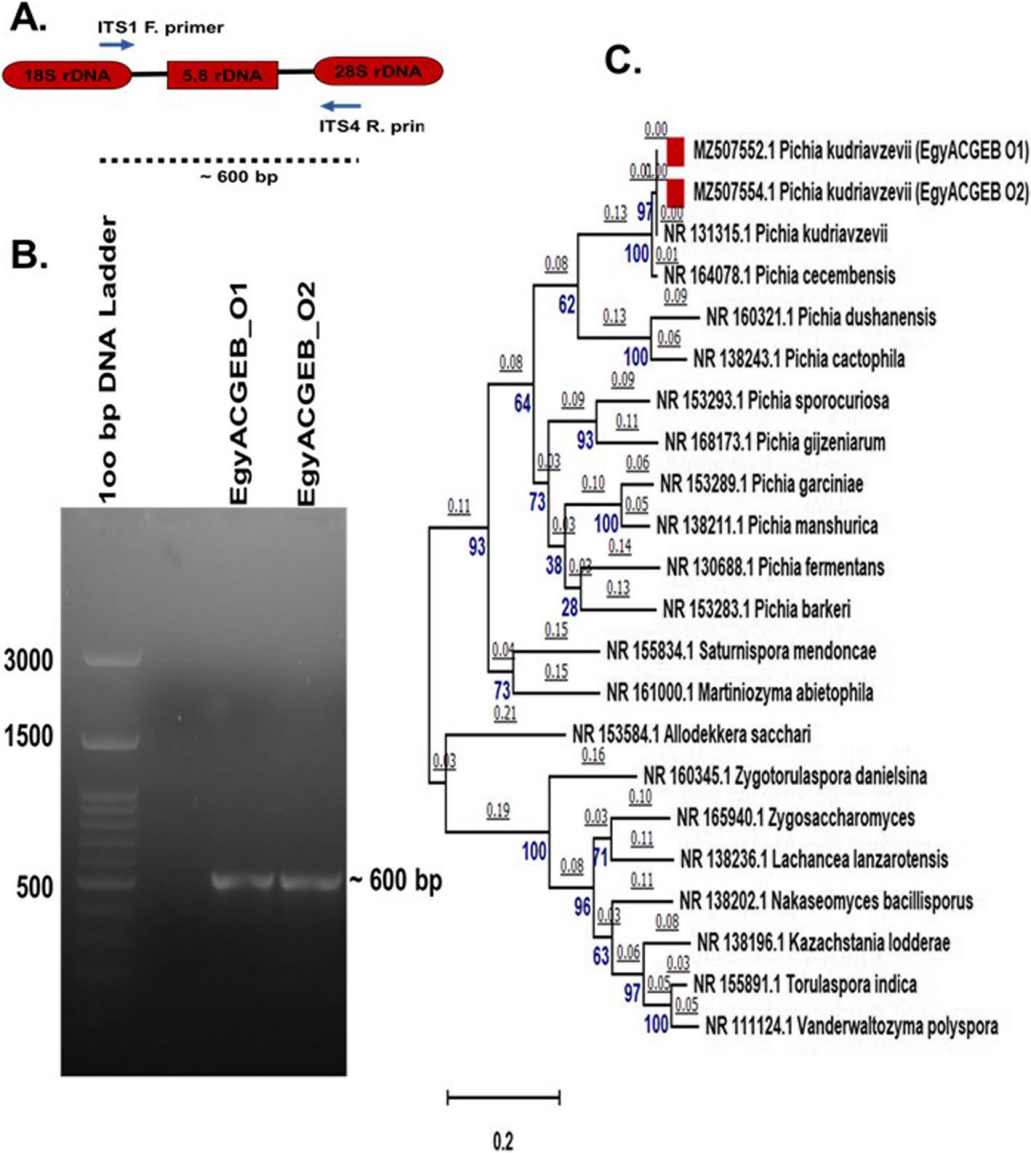
Molecular identification of EgyACGEB_O1 and EgyACGEB_O2 isolates. **A** PCR amplification of the ITS region of the genomic DNA of EgyACGEB_O1 and EgyACGEB_O2 isolates using the ITS1 and ITS4 primers covered partial part of 18S and 28S as well as 5.8S rRNA genes. **B** Agarose gel electrophoresis of PCR products showing the amplification of a ~ 600 bp fragment from both EgyACGEB_O1 and EgyACGEB_O2 isolates, original gel (Fig. S1). **C** Maximum likelihood phylogenetic tree generated using the ITS region sequences of Pk_EgyACGEB_O1, Pk_EgyACGEB_O2, and other *Pichia* species and related genera. The tree is divided into two clades (I and II) showing the evolutionary relationship between the *P. kudriavzevii* strains and other related species and genera. Evolutionary analyses were conducted in MEGAX

The genomic DNA of EgyACGEB_O1 and EgyACGEB_O2 isolates was characterised using the internal transcribed spacer (ITS) region. Especially, the sequence between the ITS1 and ITS4 genomic regions was identified by PCR amplification (Fig. 2A). This region is a highly conserved region found between the 18S, 5.8S, and 28S rRNA genes shared across many fungal species, as well as variable regions that differentiate between closely related species. After PCR amplification using the ITS1 and ITS4 primers, a fragment of 600 bp in size was obtained (Fig. 2B, Fig. S1), then sequenced. The DNA sequences of the two isolates were then compared to sequences in the GenBank NR database using the BLASTn search tool. The most similar DNA sequence to EgyACGEB_O1 and EgyACGEB_O2 isolates covered the ITS region of *P. kudriavzevii* (strain: ATCC 6258; GB: NR_131315.1) resulting in 99.8% and 99.5% DNA sequence identity, respectively. Both sequences were submitted to the GenBank database and assigned as GB: MZ507552.1 and MZ507554.1 for EgyACGEB_O1 and EgyACGEB_O2, respectively. Moving forward, we referred to them as the Pk_EgyACGEB_O1 and Pk_EgyACGEB_O2 strains.

To further explore the evolutionary relationship between Pk*_*EgyACGEB_O1 and Pk*_*EgyACGEB_O2 strains and other related species, the multiple sequence alignments by ClustalW and the evolutionary phylogenetic tree were generated using the Maximum Likelihood method (Fig. 2C). The final dataset included 22 nucleotide sequences with a total of 328 positions, including sequences from various species such as NR_131315.1 (*P. kudriavzevii*), NR_164078.1 (*P. cecembensis*), NR_160321.1 (*P. dushanensis*), NR_138243.1 (*P. cactophila*), NR_153293.1 (*P. sporocuriosa*), NR_168173.1 (*P. gijzeniarum*), NR_153289.1 (*P. garciniae*), NR_138211.1 (*P. manshurica*), NR_130688.1 (*P. fermentans*), and NR_153283.1 (*P. barkeri*), as well as other related genera. The tree analysis identified two distinct clades (I and II) that highlighted the genetic distance between *P. kudriavzevii*, other closely related *Pichia* species, and genera. First, clade I demonstrated the genetic distance between Pk*_*EgyACGEB_O1 and Pk*_*EgyACGEB_O2 strains and the closest species to *P. kudriavzevii*. Clade II, on the other hand, showed the distance with other related genera. The molecular characterization of these two *P. kudriavzevii* strains may contribute to a better understanding of their taxonomic classification. This information may be beneficial for future studies, including those related to the ecology, diversity, and evolution of these strains.

### In vitro antifungal activity of Pk*_*EgyACGEB_O1 and Pk*_*EgyACGEB_O2 strains against plant fungi

**Fig. 3:**
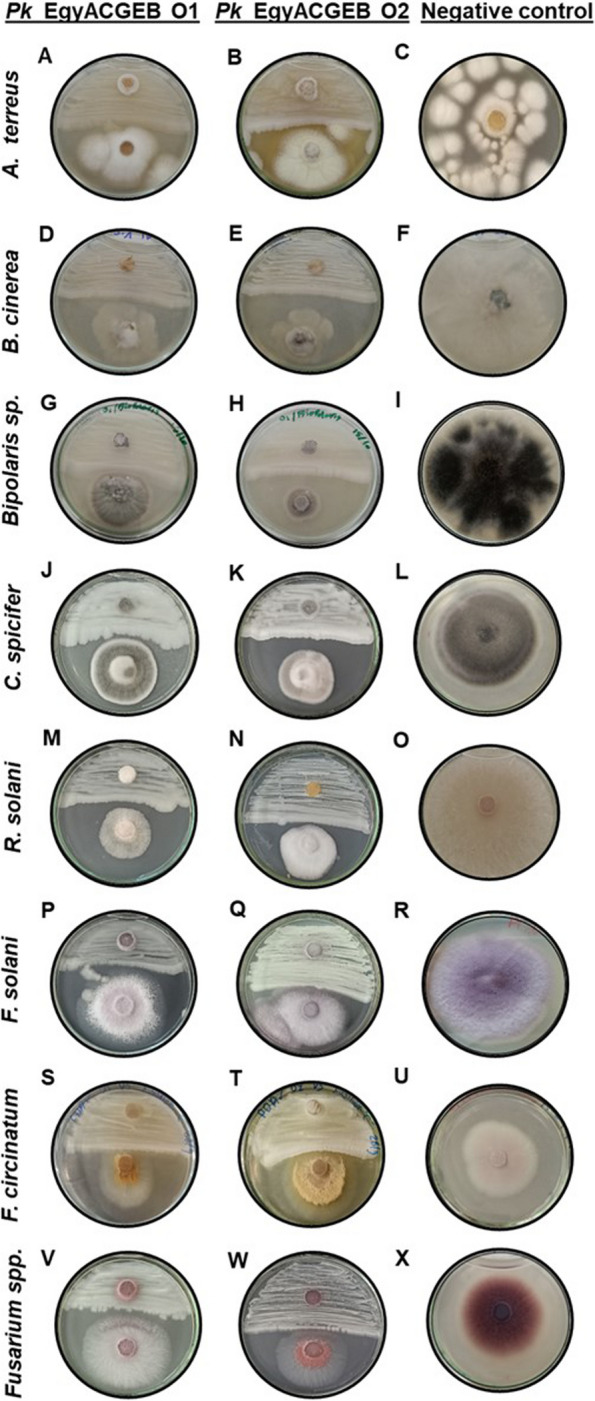
Assessment of the in vitro antagonism of Pk*_*EgyACGEB_O1 and Pk*_*EgyACGEB_O2 to plant pathogenic fungi. **A-B** The panel of plant pathogens tested included *A. terreus*, **D-E** *B. cinerea*, **G-H** *Bipolaris sp.*, **J-K** *C. spicifer*, **M–N** *R. solani*, **P-Q** *F. solani*, **S-T** *F. circinatum*, and **V-W** *Fusarium spp.* for Pk_EgyACGEB_O1 and Pk_EgyACGEB_O2, respectively; Performed three plate replicates for each fungal pathogen. Pk_EgyACGEB_O2 was found to be more effective than Pk*_*EgyACGEB_O1 in inhibiting the growth of all tested plant pathogenic fungi except for *A. terreus* that revealed a mild impact. The negative control demonstrated the growth of the fungi without any inhibitory effects: **C** *A. terreus,* **F** *B. cinereal*, **I** *Bipolaris sp.,* **L** *C. spicifer*, **O** *R. solani*, **R** *F. solani*, **U** *F. circinatum*, and **X** *Fusarium spp*

To assess the potential inhibitory effect of the Pk*_*EgyACGEB_O1 and Pk*_*EgyACGEB_O2 strains on various plant pathogenic fungi, an in vitro screening experiment on Petri dishes containing Potato Dextrose Agar (PDA) medium over 14 days was conducted. The panel of plant pathogens tested in this experiment included *A. terreus, B. cinerea, Bipolaris sp., C. spicifer, R. solani, F. solani, F. circinatum*, and *Fusarium spp*. that are known to cause significant damage to crops, vegetables, and fruits. In three replicates, the Pk*_*EgyACGEB_O1 or Pk*_*EgyACGEB_O2 strain was streaked on PDA medium agar plates, covering only one-half of the plate, leaving the other half as a control. The growth of each fungal pathogen that was inoculated on a disk positioned on one half of the plate was monitored and compared to the control, the unstreaked area. For the negative control, we positioned each fungal-inoculated disk in the middle of the PDA medium agar plate, allowing normal growth. Overall, both Pk_EgyACGEB_O1 and Pk_EgyACGEB_O2 strains have the potential to inhibit the plant pathogenic fungi at various levels compared to control (Fig. 3A-X). In addition, Pk_EgyACGEB_O2 was found to be more effective than Pk_EgyACGEB_O1 in inhibiting the growth of all the tested plant pathogenic fungi, except for *A. terreus*, which showed only a mild impact (Fig. 3B).

**Fig. 4:**
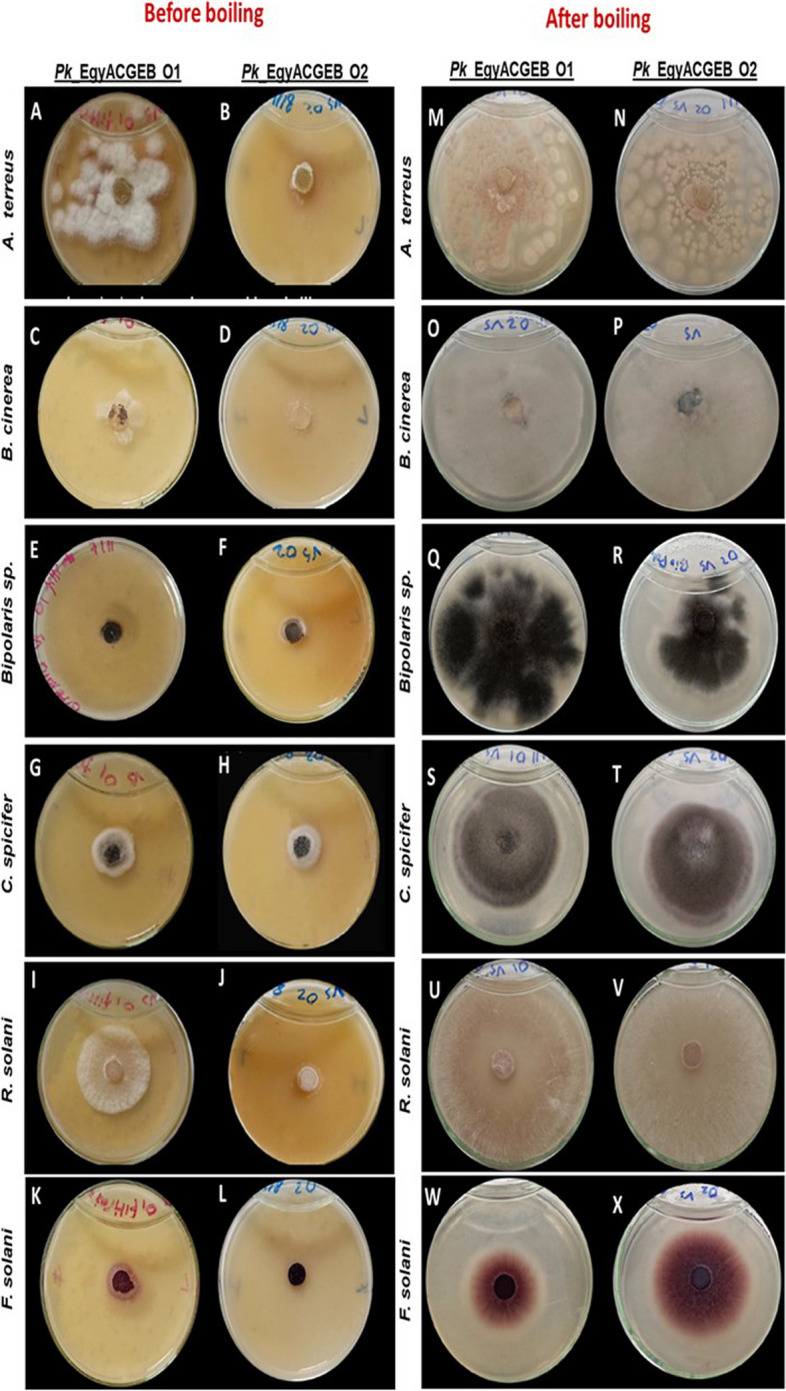
In vitro assessment of Pk*_*EgyACGEB_O1 and Pk*_*EgyACGEB_O2 filtrates against plant fungi. The culture filtrate of both strains consistently inhibited the growth of all tested fungi including *A. terreus, B. cinerea, Bipolaris spp., C. spicifer, R. solani*, and *F. solani* (**A** to **L**); Performed three plate replicates for each fungal pathogen. However, Pk*_*EgyACGEB_O1 filtrate had a slight impact on *A. terreus* and *R. solani* (**A** and **I**). Boiling the culture filtrates for five minutes reduced the inhibitory impact of both strains on the pathogenic fungi (**M** to **X**)

The study raised a question about the mode of action of the antifungal activity of Pk_EgyACGEB_O1 and Pk_EgyACGEB_O2 strains, whether it is triggered by fungi or if they consistently secrete inhibitor enzymes. To understand this, two additional experiments were conducted. In the first experiment, the efficacy of the extracellular secreted components of each strain was assessed by preparing a filtrate of a five-day YMPG growth culture of each strain mixed with PDA agar medium in a 1:4 volume ratio (as described in the methods). The culture filtrates of both strains, especially Pk_EgyACGEB_O2, consistently inhibited the growth of all tested fungi: *A. terreus, B. cinerea, Bipolaris spp., C. spicifer, R. solani,* and *F. solani* (Fig. 4A-L). However, Pk_EgyACGEB_O1 filtrate had a slight impact on *A. terreus* and *R. solani* (Fig. 4A-I). On the other hand, we verified that inhibition of fungi by the secreted components of Pk_EgyACGEB_O1 and Pk_EgyACGEB_O2 strains was due to the presence of proteins by boiling Pk_EgyACGEB_O1 and Pk_EgyACGEB_O2 filtrates. We found that boiling the filtrates for five minutes reduced the inhibitory impact of both strains on the pathogenic fungi (Fig. 4M-X). Overall, these results provided insights into the action of the antifungal activity of Pk*_*EgyACGEB_O1 and Pk*_*EgyACGEB_O2. The consistent inhibition of the tested fungi by the culture filtrates of both strains suggests that they secrete antifungal compounds, possibly proteins, which could be responsible for their antifungal activity.

### *Pichia kudriavzevii* as a potential agent for post-harvest preservation

**Fig. 5:**
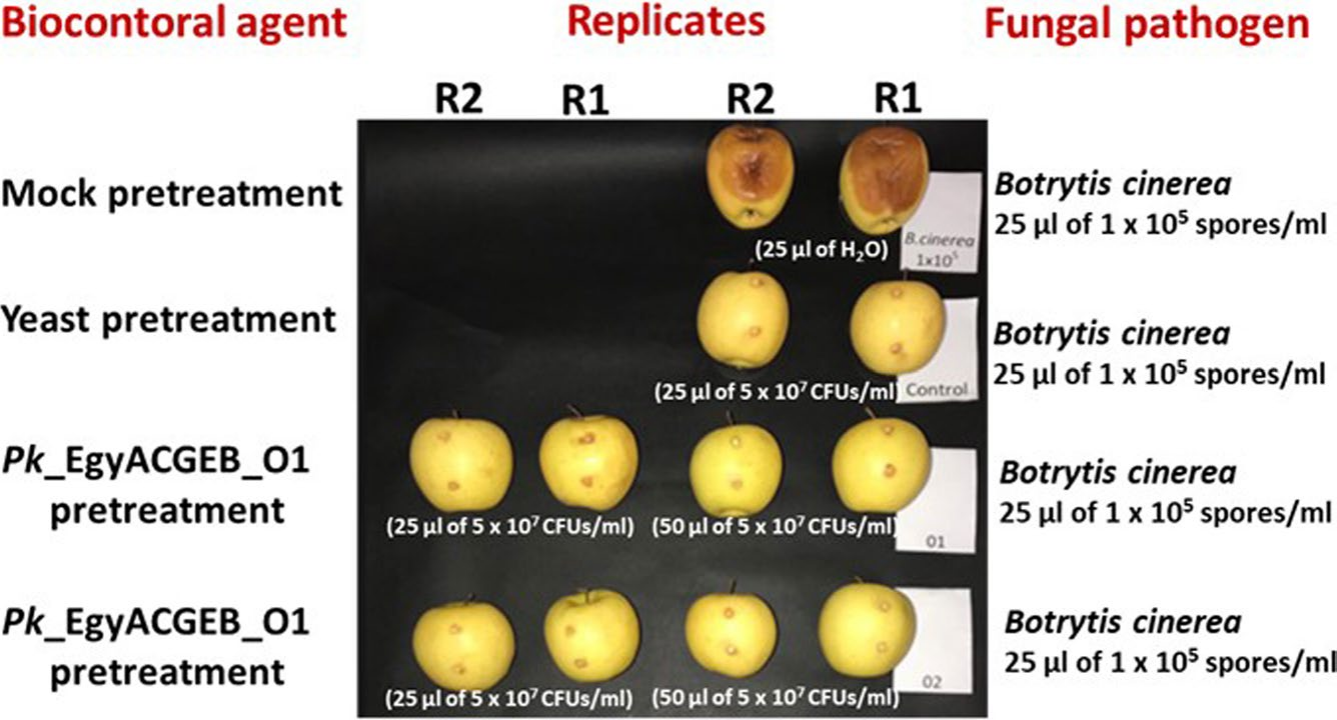
The effectiveness of Pk*_*EgyACGEB_O1 and Pk*_*EgyACGEB_O2 in controlling *Botrytis cinerea* infecting apples. Golden delicious apples were pretreated with either Pk*_*EgyACGEB_O1 or Pk*_*EgyACGEB_O2 at concentrations of 25 µl of 5 × 10^7^ CFUs/ml or 50 µl of 5 × 10^7^ CFUs/ml, respectively, and then inoculated with *B. cinerea* at a concentration of 25µl of 1 × 10^5^ spores/ml. After 21 days, apples pretreated with either Pk*_*EgyACGEB_O1 or Pk*_*EgyACGEB_O2 exhibited significant efficacy in controlling gray mold disease, as evidenced by the reduced incidence of illness and lesion width. A higher concentration of Pk*_*EgyACGEB_O1 or Pk*_*EgyACGEB_O2 pretreatment led to better disease control efficacy indicating a dose–response relationship

Both *P. kudriavzevii* strains, Pk_EgyACGEB_O1 and Pk_EgyACGEB_O2, have demonstrated the ability to effectively suppress the growth of *Botrytis cinerea*, the most important post-harvest apple disease that causes gray mold. For assessment, golden delicious apples were pretreated with *Pichia* strains, then inoculated with *B. cinerea* (details described in the methods)*.* For comparison, we used the same concentration of activated commercial yeast and water as positive and negative pretreatment controls. After a period of 21 days, we observed that pretreated apples with either Pk*_*EgyACGEB_O1 or Pk*_*EgyACGEB_O2 exhibited notable efficacy in controlling gray mold disease (Fig. 5, Table 1). In contrast, mock-treated apples (negative control) displayed a higher lesion width (62 mm), indicating the presence of infection. Interestingly, a higher volume of Pk*_*EgyACGEB_O1 or Pk*_*EgyACGEB_O2 pretreatment (50 µl from 5 × 10^7^ CFUs/ml) led to better disease control efficacy when compared to a lower volume (25 µl of 5 × 10^7^ CFUs/ml). However, even lower concentrations of the *Pichia* strains still provided a considerable level of protection against *B. cinerea*. The volume of 25 µl from Pk_EgyACGEB_O1 showed a notable level of decay, resulting in lesions with an average diameter of 2.5 mm. In contrast, at the same concentration, Pk_EgyACGEB_O2 did not develop any lesions. This study provides a foundation for further research into the optimal application method and dosage for *Pichia*-based biocontrol agents and underscores the importance of sustainable and eco-friendly solutions for post-harvest diseases.

**Table 1:** Quantifying the impact of Pk_EgyACGEB_O1 and Pk_EgyACGEB_O2 inoculation on *Botrytis cinerea* that causes gray mold on the golden delicious apple cultivar

**Isolates**	**Pk_EgyACGEB_O1**		**Pk_EgyACGEB_O2**	
**Dose & concentration (CFUs/ml)**	**Lesion diameter mm**	^f^**Infected Area mm**^**2**^	**Lesion diameter mm**	^f^**Infected Area mm**^**2**^
**25 μl 5 × 10**^**7**a^	2.5^e^	10	0.0	0.0
**50 μl 5 × 10**^**7**b^	0.0	0.0	0.0	0.0
**Control P** ^c^	0.0	0.0	0.0	0.0
**Control N** ^d^	62.0	2934	62.0	2934

^**a**^Wounded golden delicious apple fruits were inoculated with 25 µl 5 × 10^7^ CFUs/ ml *Pichia* cells and challenged 60 min later with 25 μl 1 × 10^**5**^ conidia/ml of *B. cinerea*

^**b**^Wounded golden delicious apple fruits were inoculated with 50 µl 5 × 10^7^ CFUs/ ml *Pichia* cells and challenged 60 min later with 25 μl 1 × 10^**5**^ conidia/ml of *B. cinerea*

^**c**^Wounds of golden delicious apple fruits inoculated with 25 µl 5 × 10^7^ CFUs/ ml of commercial yeast cells and used as positive control

^**d**^Wounds of golden delicious apples treated with 25 µl of sterile distilled water and 60 min later inoculated with 25 µl 1 × 10^5^ conidia/ml of *B. cinerea* and used as negative control

^e^The average calculated from three fruit replicates

^f^Assessment of the infected Area (mm2) after 21 days of treatment

### The core secretome of *P. kudriavzevii*

**Fig. 6:**
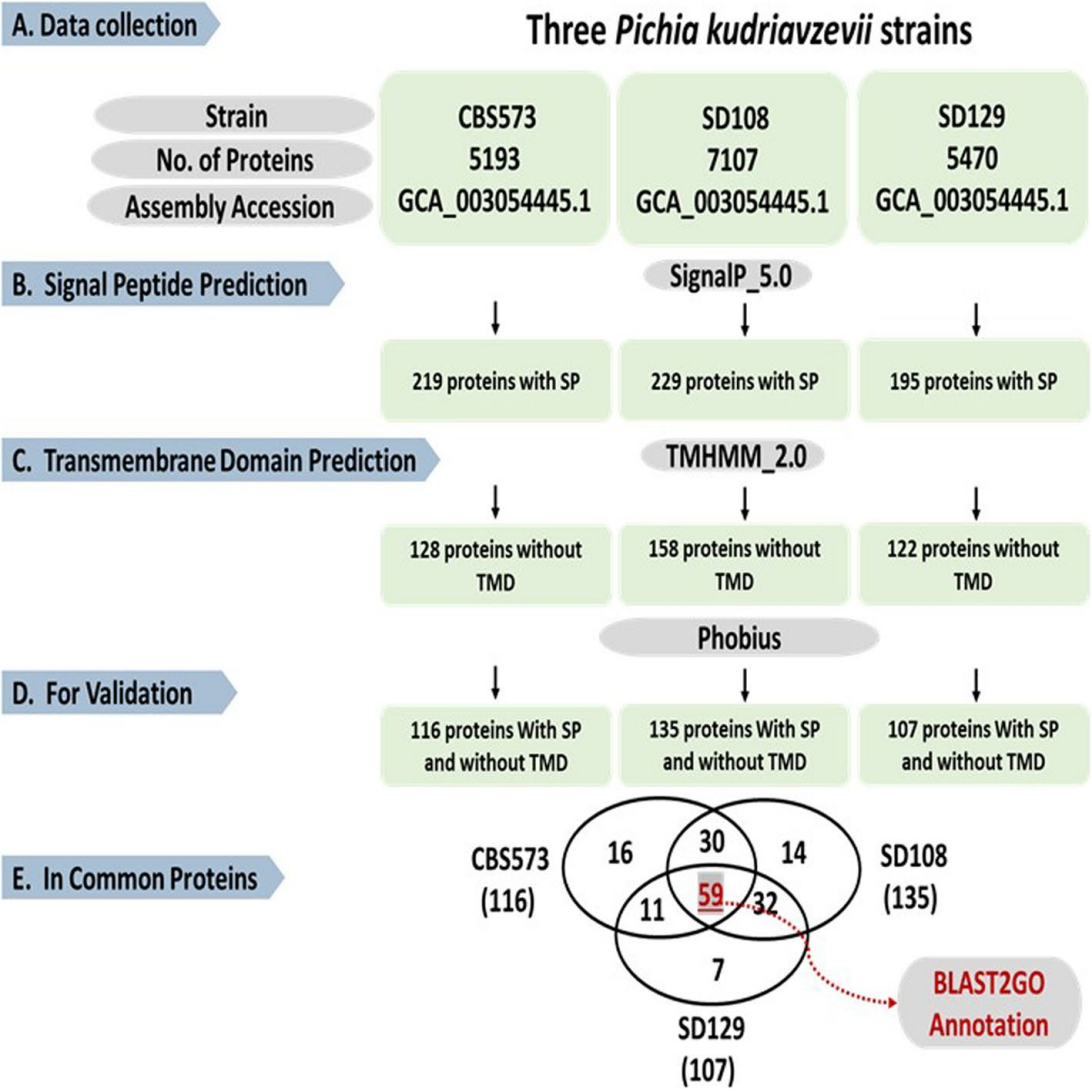
The workflow for predicting candidate-secreted proteins of *P. kudriavzevii.* **A** The total protein sequences of *P. kudriavzevii* strains CBS573, SD108, and SD129 were collected from the GenBank database. **B** Effector prediction pipeline involved signal peptide and **C** transmembrane domains prediction using SignalP and TMHMM-2.0, receptively. **D** The presence of signal peptides and absence of transmembrane domains of all filtrated proteins were validated using Phobius. **E** The number of shared and unique potential secretion proteins were identified for each strain. In common candidate-secreted proteins were functionally annotated using the Blast2GO suite

**Fig. 7:**
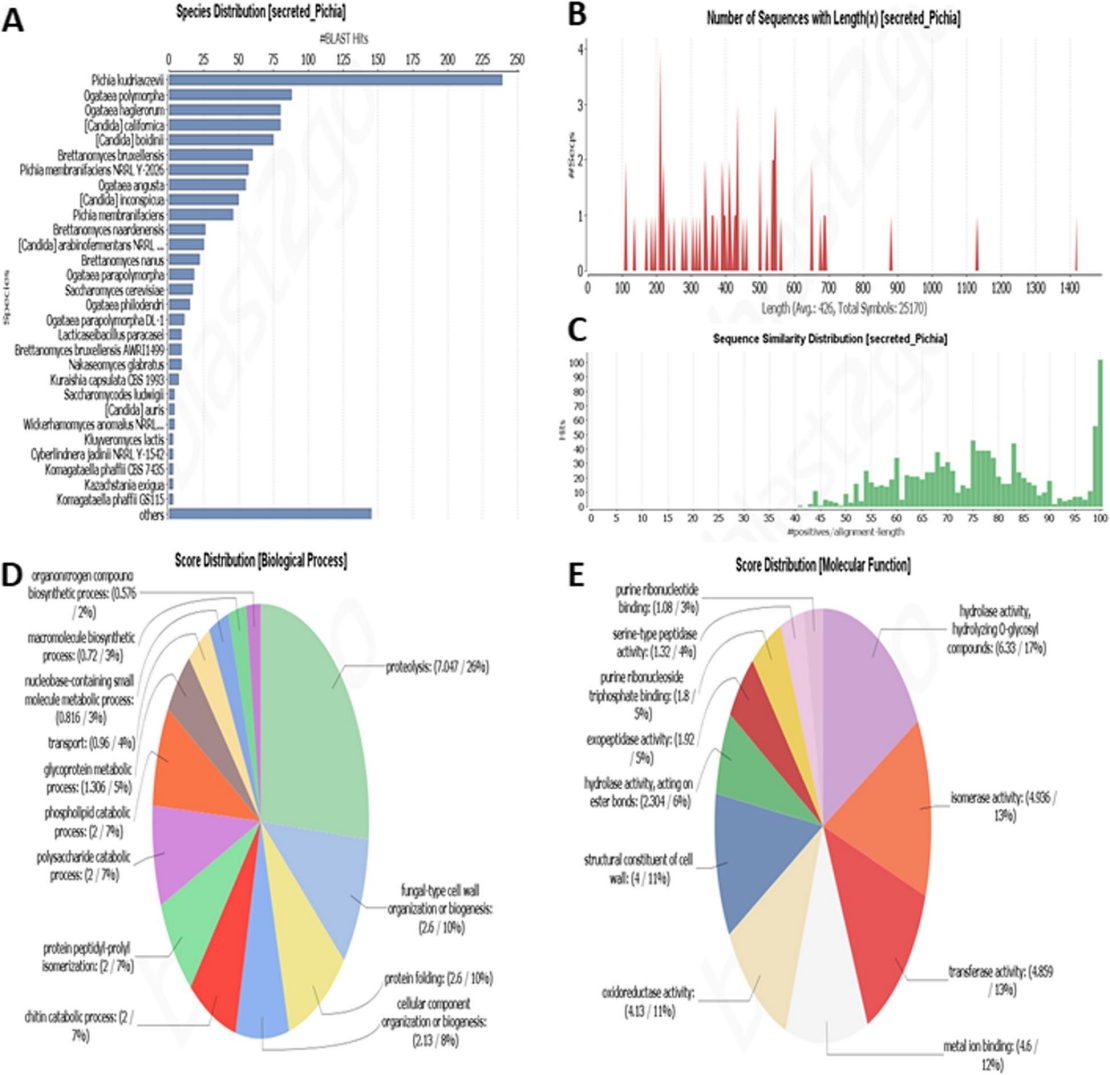
The annotation of the potential secreted proteins in *P. kudriavzevii*. **A** The majority of the identified proteins were present in *P. kudriavzevii*. **B** The identified proteins were relatively short, with less than 700 amino acid. **C** Distribution of similarity of these proteins, with an average similarity of approximately 80%. **D** Comprehensive classification of biological processes revealed that secreted proteins were involved in a diverse range of processes, including proteolysis, cell-wall biogenesis, protein folding, catabolism, isomerization, glycoprotein metabolism, and transport. **E** Molecular function of secreted proteins was categorized based on their specific biochemical activities into O-glycosyl hydrolase, isomerase, transferase, oxireductase, ester bond hydrolase, exopeptidase, and serine-type peptidase

To identify the essential proteins responsible for *P. kudriavzevii* potent antifungal activity, we employed a comprehensive bioinformatics pipeline to predict the secretion proteins of three distinct *P. kudriavzevii* strains, CBS573, SD108, and SD129. These strains were selected based on their genomic availability in GenBank. The bioinformatics pipeline was designed to ensure consistency and comparability of the results for each strain analysed (Fig. 6). The pipeline involved several computational tools to predict the potential secretion proteins based on their signal peptides and transmembrane domains (as described in the methods). Proteins exhibiting signal peptides and lacking transmembrane domains were chosen as candidates displaying secretion capability (Fig. 6 B, C). The presence of signal peptides and the absence of transmembrane domains of all filtrated proteins were validated using Phobius, a tool to predict the signal peptide and TM domain (Fig. 6D). Through this filtration process, we identified a total of 358 potential secretion proteins, which may play a crucial role in the antifungal activity of *P. kudriavzevii*. Among these proteins, we found 59 proteins common to all three strains that may play a fundamental role in the *P. kudriavzevii* secretome (Fig. 6E). In addition, we identified 16 proteins unique to CBS573, 14 unique to SD108, and 7 unique to SD129. Approximately 53 out of 59 candidate-secreted proteins were successfully functionally identified and classified into distinct categories based on their functions (Table 2). These proteins were classified into different functional categories, which encompassed enzymes (31 proteins), cell-wall-related proteins (9), inhibitors (1), and other types of proteins (12). To provide valuable insights into their characteristics and functions, the full annotation of candidate-secreted proteins was performed using the blast2GO suite. The majority of the proteins were present in *P. kudriavzevii* (Fig. 7A). In addition, these proteins were relatively short, with less than 700 amino acids (Fig. 7B). The distribution of the similarity of these proteins has an average of approximately 80% (Fig. 7C).

**Table 2:** The identification of *P. kudriavzevii* potential secreted proteins

**Classification**	**ID**	**Description**	**Protein length**	**Enzyme code**
Various enzymatic proteins	ONH70544.1	3-phytase A	542	EC:3.1.3.8
	ONH69279.1	3-phytase B	542	EC:3.1.3.8
	ONH72606.1	1,3-beta-glucanosyltransferase gas1	549	EC:2
	ONH74370.1	Aminopeptidase Y	504	EC:3.4.11
	ONH77310.1	AScCTS1_apo crystal structure	537	EC:3.2.1
	ONH77655.1	Aspartic proteinase 3	562	EC:3.4.23
	ONH71381.1	Carboxypeptidase Y	549	EC:3.4.16
	ONH71456.1	Carboxypeptidase Y	546	EC:3.4.16
	ONH75632.1	Cell surface mannoprotein MP65	394	EC:3.2.1
	ONH75618.1	Chitin deacetylase 2	318	EC:3.5
	ONH77345.1	Cytochrome b2, mitochondrial precursor	188	EC:1
	ONH77133.1	D-amino-acid oxidase	366	EC:1.4.3.3
	ONH77760.1	Endochitinase 1	461	EC:3.2.1
	ONH74881.1	ER degradation-enhancing alpha-mannosidase-like protein 1	1130	EC:2.7.1
	ONH77291.1	ERAD-associated protein	882	EC:6
	ONH77673.1	FK506-binding protein 2	138	EC:5.2.1.8
	ONH72980.1	Flocculation protein FLO11	343	EC:3.6.1
	ONH75975.1	Glucan 1,3-beta-glucosidase	428	EC:3.2.1
	ONH70941.1	Hydrolase activity, acting on glycosyl bonds protein	430	EC:3
	ONH70264.1	L-saccharopine oxidase	437	EC:1
	ONH74522.1	Lysophospholipase 1	653	EC:3.1.1.5
	ONH77577.1	Lysophospholipase 1	677	EC:3.1.1.5
	ONH75482.1	Peptidase M20 domain-containing protein	416	EC:3.6.1
	ONH76622.1	Peptidyl-prolyl cis–trans isomerase B	213	EC:5.2.1.8
	ONH72590.1	protein disulfide isomerase family A, member 6	394	EC:5
	ONH76889.1	Protein disulfide-isomerase precursor	522	EC:5.3.4.1
	ONH77299.1	proteinase B	538	EC:3.4.21
	ONH77575.1	UDP-glucose:glycoprotein glucosyltransferase	1423	EC:2.4.1
	ONH72438.1	Putative secreted beta-glucosidase adg3	306	
	ONH76893.1	Glucosidase 2 subunit beta	502	
	ONH77163.1	Beta-glucosidase-like protein NCA3, mitochondrial	378	
**Cell-wall related proteins**	ONH70361.1	Cell wall mannoprotein PIR1	328	
	ONH70474.1	Cell wall protein PRY3	343	
	ONH74140.1	Cell wall mannoprotein PIR1	275	
	ONH74903.1	Cell wall protein ECM33	436	
	ONH77079.1	Cell wall protein RHD3	210	
	ONH77080.1	Cell wall protein RHD3	210	
	ONH74281.1	Yeast-form wall Protein 1	435	
	ONH74315.1	Yeast-form wall Protein 1	399	
	ONH72487.1	Pir1 1,3-beta-glucan-linked structural cell wall protein	347	
**Papain inhibitor**	ONH76293.1	Papain inhibitor	220	
**Various function proteins**	ONH71108.1	CFEM protein	223	
	ONH74416.1	Chaperone protein DnaJ	653	
	ONH73171.1	Clock-controlled protein 6	110	
	ONH76423.1	Flocculation protein FLO10	693	
	ONH74939.1	GPI-anchored protein 52	404	
	ONH72668.1	Mating factor alpha precursor N-terminus	217	
	ONH75639.1	Protein PRY1	253	
	ONH77573.1	Purine nucleoside permease	411	
	ONH76325.1	Putative secreted protein	114	
	ONH71839.1	Synchronized import protein 1	687	
	ONH74326.1	Temperature shock-inducible protein 1	213	
	ONH73001.1	Tos1p	451	
**Hypothetical protein**	ONH71006.1	Hypothetical protein BOH78_4805	172	
	ONH72010.1	Hypothetical protein CAS74_000109	288	
	ONH72787.1	Hypothetical protein JL09_g3193	238	
	ONH75128.1	Hypothetical protein BOH78_2009	363	
	ONH76839.1	Hypothetical protein BOH78_0716	198	
	ONH77587.1	Hypothetical protein BOH78_0357	411

Furthermore, to gain a better understanding of the roles of these potential secretion proteins in biological systems, we performed a comprehensive classification of their biological processes. The results revealed that candidate-secreted proteins were involved in a diverse range of biological processes, including proteolysis, which accounted for 26% of the proteins, cell-wall biogenesis (10%), protein folding process (10%), cellular component organization (8%), chitin, polysaccharide, and phospholipid catabolism (21%), peptidyl-prolyl isomerization (7%), glycoprotein metabolism (5%), and transport process (4%) (Fig. 7D). Additionally, we precisely dissected the molecular function of these secreted proteins and categorized them based on their specific biochemical activities into O-glycosyl hydrolase (17%), isomerase (13%), transferase (13%), oxireductase (11%), ester bond hydrolase (6%), exopeptidase (5%), and serine-type peptidase (4%) (Fig. 7E). Overall, the results of our study provided insights into using bioinformatics tools to predict potential secretion proteins of *P. kudriavzevii*. These findings may facilitate the development of new antifungal agents and offer valuable information for future research in this field. Furthermore, our study demonstrates the importance of using bioinformatics tools and algorithms to predict potential secretion proteins, which may speed up the discovery of novel proteins and their roles in various biological processes.

## Discussion

Biocontrol agents are typically safer for the environment and human health, as they do not leave harmful residues in the soil or on the plants. They can also be more cost-effective in the long run, as they can help to reduce the need for repeated fungicide applications. Biocontrol agents can be an effective and sustainable way to control fungal diseases in plants [10, 12]. The development of biocontrol agents from *Pichia* represents potential for a highly effective approach to controlling fungal diseases in plants that can provide a range of benefits for both farmers and the environment [15, 17]. Many species of *Pichia* have antagonistic activity against a range of plant pathogens, including fungi [16, 17, 22, 23]. We investigated two novel *P. kudriavzevii* strains, Pk_EgyACGEB_O1 and Pk_EgyACGEB_O2, GenBank accession numbers: MZ507552.1 and MZ507554.1, respectively. The molecular identification of both strains using the ITS region provided a valuable contribution to understanding their genetic makeup. The most similar DNA sequence for both isolates covered the ITS region of *P. kudriavzevii*. The generated phylogenetic tree provides a visual representation of the evolutionary relationships between Pk_EgyACGEB_O1 and Pk_EgyACGEB_O2 strains with the most similar *Pichia* species and other similar genera. The two clades observed in the tree suggest significant genetic differences between *P. kudriavzevii*, the genus *Pichia*, and other similar genera. The ITS region is highly conserved among fungal species, but it also contains variable regions that can be used to differentiate between closely related species [30]. By comparing the ITS sequences of different species of *Pichia*, researchers can identify new species or clarify the taxonomic status of existing ones. This can help improve our understanding of the fungal kingdom and aid in developing more accurate and comprehensive classification systems. This information can be useful for studying the development of different yeast species, understanding how they have evolved, and monitoring the prevalence and distribution of *Pichia* in different environments.

The in vitro screening of Pk*_*EgyACGEB_O1 and Pk*_*EgyACGEB_O2 strains against plant pathogenic fungi is essential to illuminate their potential to control plant fungal diseases. Interestingly, Pk_EgyACGEB_O2 was more effective than Pk_EgyACGEB_O1 in inhibiting the growth of all tested plant pathogenic fungi including, *A. terreus*, *B. cinerea*, *Bipolaris sp*., *C. spicifer*, *R. solani*, *F. solani*, *F. circinatum*, and *Fusarium spp.* This is a significant finding, as it suggests that Pk_EgyACGEB_O2 could be a valuable tool in controlling plant diseases. Furthermore, these findings may pave the way for developing novel strategies to combat plant pathogens and increase crop yields. Several *Pichia* species have been shown to have the potential to control a range of plant pathogens through various mechanisms such as competition for nutrients [14, 16], production of antifungal metabolites [19], and induction of host plant defence mechanisms [18]. In addition, we pursued understanding the mode of action of the antifungal activity of the Pk_EgyACGEB_O1 and Pk_EgyACGEB_O2 strains. First, we assessed the effectiveness of Pk*_*EgyACGEB_O1 and Pk*_*EgyACGEB_O2 strain filtrates. The results showed that both strains consistently secrete inhibitor proteins, as their filtrates prevent the growth of most tested fungi. Boiling the *Pichia*-YMPG filtrates for reducing the inhibitory impact of the two tested isolates on the pathogenic fungi. This further supports the hypothesis that the inhibitory effect of the strains is due to the secretion of enzymes. This also provides valuable insight into the mechanisms that enable these strains to impede fungal growth and could pave the way for future research on their possible uses in various industries.

Post-harvest losses due to fungal infections are a significant challenge for the agricultural industry, resulting in a waste of resources and economic losses for farmers. The use of synthetic fungicides is a standard solution, but it raises concerns about food safety and environmental sustainability. Therefore, there is a growing interest in developing alternative eco-friendly solutions for post-harvest disease management [10]. In this context, we investigated the impact of *P. kudriavzevii* as a post-harvest preservation agent, which is a significant step forward in finding a sustainable and effective solution for fungal control in fruits. The study demonstrates that *P. kudriavzevii* strains, Pk*_*EgyACGEB_O1 and Pk*_*EgyACGEB_O2, have significant potential for controlling *Botrytis cinerea,* a major post-harvest disease in the golden delicious apple cultivar (Fig. 5). The study shows that the use of *Pichia* strains as a pretreatment method can lead to effective control of gray mold disease, which is crucial in reducing post-harvest losses. Furthermore, the study indicates that a higher concentration of *Pichia* strains leads to better disease control efficacy, highlighting the importance of optimising the application method and dosage. Two studies investigated the potential of *P. kudriavzevii* as a biocontrol agent to reduce fungal decay in cherry tomatoes [22] and gray mold disease in grapevines [23]. Overall, our study and other related research have yielded promising results and warrant further exploration into utilising *P. kudriavzevii* strains for post-harvest fruit preservation.

The biocontrol activity of *P. kudriavzevii* is due to the secretion of various proteins, including enzymes, cell wall proteins, antimicrobial peptides, and other bioactive molecules. The identification and evaluation of these secreted proteins are crucial for understanding the biocontrol activity of *Pichia*. The significance of predicted secreted proteins for the biocontrol activity of *P. kudriavzevii* lies in the fact that these proteins can be potential targets for developing new biocontrol strategies against plant pathogens. A comprehensive analysis of the predicted secretome of *P. kudriavzevii* revealed that approximately 23% of the predicted secretome is composed of broad-spectrum hydrolases acting on both O-glycosyl compounds and ester bonds, such as proteases, lipases, glycosidases, phosphatases, esterases, carboxypeptidases, and peptidases (Fig. 7E; Table 2). These hydrolase enzymes degrade a wide range of substrates including, cell walls, proteins, and lipids, which are essential components of many plant pathogens. Many species of *Pichia* have shown promise as a source of diverse and versatile hydrolase enzymes, such as proteases generated by *P. pastoris* [31], lipases produced by *P. pastoris* and *P. guilliermondii* [32, 33] and peptidases expressed by *P. anomala* and *P. pastoris* [34, 35]. As a result, these enzymes can potentially disrupt the cellular integrity of plant pathogens and reduce their virulence. In general, biocontrol agent enzymes possess recognition sites or binding domains that are specific to certain protein structures found in fungal proteins. This specificity enables them to selectively target and degrade fungal proteins while leaving plant proteins unaffected. One example of an enzyme that targets fungal cell walls is chitinase. Chitinase is an enzyme that specifically degrades chitin, a major component of fungal cell walls [36]. Chitin is absent in plant cell walls, making chitinase highly selective in targeting fungal pathogens while sparing plant tissues. This selective action of chitinase on fungal cell walls provides an effective means of controlling fungal diseases while minimizing any impact on plant tissues. Biocontrol agent enzymes may exhibit optimal activity under specific environmental conditions, such as pH or temperature, that are characteristic of the fungal habitat [37]. This can further enhance their ability to selectively target and degrade fungal proteins while maintaining stability in the presence of plant proteins. Another class of proteins identified is the cell-wall-related proteins such as cell-wall mannoprotein PIR1, cell-wall protein PRY3, RHD3, and ECM33, which play a crucial role in maintaining the structure and integrity of the cell wall for cell growth and division. Previously, a study demonstrated the importance of PIR1 in forming biofilms [38]. PIR1 contributes to the formation of biofilms by promoting cell–cell adhesion and binding to the extracellular matrix. Furthermore, papain inhibitors may provide additional protection against plant pathogens. Papain is a plant protease that is involved in many physiological processes, and plant pathogens have been shown to produce and secrete papain as a virulence factor [39]. We suggest that *P. kudriavzevii* may be able to provide additional protection to host plants against papain-secreting pathogens by preventing them from causing damage to the host plant. Overall, the identification of these potentially secreted proteins from *P. kudriavzevii* provides valuable insight into the mechanisms by which this species of *Pichia* can act as a biocontrol agent against plant pathogens. Further expression studies on this set of proteins could lead to the development of new and effective strategies for controlling plant diseases.

## Conclusions

The potential of using novel *Pichia kudriavzevii* strains, Pk*_*EgyACGEB_O1 and Pk*_*EgyACGEB_O2, as biocontrol agents to combat fungal diseases in plants has been investigated. The morphological and genetic characteristics of *P. kudriavzevii* strains were identified. We found that Pk*_*EgyACGEB_O2 was more effective than Pk*_*EgyACGEB_O1 in inhibiting the growth of plant pathogenic fungi. The potential of *P. kudriavzevii* strains as a post-harvest preservation agent to control *B. cinerea*, a major post-harvest disease in the golden delicious apple cultivar. The use of *Pichia* strains as a pretreatment method can lead to effective control of gray mold disease, which is crucial in reducing post-harvest losses. The secreted proteins of *P. kudriavzevii* have significant potential for biocontrol and biotechnological applications. They include enzymes with hydrolysis, proteolysis, and lipase activity, which can degrade cell walls and disrupt the cellular integrity of plant pathogens. These predicted secretomes can be utilised for developing new biocontrol strategies against plant pathogens and for the production of enzymes and bioactive molecules. The biocontrol activity of *P. kudriavzevii* is attributed to the secretion of various proteins, including hydrolases, cell wall-related proteins, and papain inhibitors. The predicted secreted proteins have the potential to be used as targets for developing new biocontrol strategies against plant pathogens. The identification and evaluation of these proteins provide valuable insight into the mechanisms by which *P. kudriavzevii* can act as a biocontrol agent against plant pathogens, and further studies on this set of proteins could lead to the development of new and effective strategies for controlling plant diseases.

### Perspective

*P. kudriavzevii* shows promising potential as a biocontrol agent that can inhibit the growth of various plant pathogens. However, the mechanisms behind its biocontrol activity are not fully understood. Further research is needed to improve our understanding of the biocontrol mechanisms of *Pichia*, which can lead to the development of more effective biocontrol strategies. One area of research that can improve our understanding of the biocontrol mechanisms of *P. kudriavzevii* is the expression of the secreted candidate genes involved in their biocontrol activity. While bioinformatics tools have been used to predict the secretomes of *P. kudriavzevii*, experimental validation of these predictions is necessary to determine the specific proteins that contribute to their biocontrol activity. Another area of research that can improve our understanding of the biocontrol mechanisms of *P. kudriavzevii* is the identification of the signalling pathways that regulate their biocontrol activity. It is known that the biocontrol activity of *Pichia* is regulated by various environmental factors, such as pH, temperature, and nutrient availability. The identification of the signalling pathways that mediate these responses can provide insight into the molecular mechanisms that govern the biocontrol activity of *Pichia*. Furthermore, research on the interaction between *P. kudriavzevii* and plant pathogens can provide insight into biocontrol mechanisms. For example, identifying the receptors on the surface of plant pathogens that interact with *P. kudriavzevii* secreted proteins can provide insight into the molecular mechanisms that underlie the inhibition of pathogen growth.

There is also a need for more comprehensive studies on the ecology of *P. kudriavzevii* in natural environments. Understanding the natural habitats of *P. kudriavzevii* and how they interact with other microorganisms can provide insight into their potential as biocontrol agents in agricultural settings. The potential of the selected *Pichia* isolates as biocontrol agents will depend on several factors, such as their specificity, compatibility with other agents, and environmental conditions. To overcome the challenges associated with the use of biocontrol agents, several potential solutions can be considered. For instance, developing a consortium of biocontrol agents that are more effective under a broader range of environmental conditions can enhance their efficacy. Using complementary microorganisms can also enhance the efficacy of biocontrol agents and promote their use in sustainable crop protection programs.

In addition to the above perspectives, developing formulations that can improve the stability and efficacy of *P. kudriavzevii* as a biocontrol agent is also an important area of research. Formulations such as microencapsulation or nanotechnology-based delivery systems can protect the biocontrol agent from environmental stressors and increase its shelf life, allowing for more effective and efficient use in agricultural settings.

## Methods

### Sampling strategy

This study aimed to isolate various strains of *Pichia*. The olives used in the experiment were of the picual cultivar (*Olea europaea*) and subjected to a six-month fermentation process in a 10% (w/v) NaCl brine solution. We collected triplicate samples from the fermented brines. To ensure comprehensive sampling, triplicate samples were obtained from the fermented brines, derived from two different locations. Samples of olive brine were collected and transferred aseptically to the lab under sterile conditions to prevent contamination. About 1 ml from each sample was added into the YMPG broth medium containing 3 g/l yeast extract, 3 g/l malt extract, 5 g/l peptone, and 10 g/l glucose, then incubated at 25 °C for 3 days to allow the microorganisms to multiply. For dilution, 10 ml from each incubated culture was inoculated in 90 ml of distilled-sterilised water, then diluted by serial dilutions. On YMPG agar plates, 1 ml of each dilution was dispersed and incubated at 25 °C for 3 days. Only single colonies were selected, purified, and stored at -20 °C in sterile 70% glycerol.

### Phenotypic identification

To morphologically identify selected *Pichia* isolates, purified colonies were subjected to standard classification schemes as described by Kreger-van Rij [29]. In brief, the classification involved examining the morphology of the cells and their ability to produce spherical, ascospore, and pseudohyphae formations by the light microscope and crystal violet stain (1g crystal violet powder dissolved in 20% methanol).

### Molecular identification

#### DNA isolation

Single colonies were collected and inoculated into 2 ml of YMPG media. The cell pellets from 1.5 ml of the overnight cultures were resuspended in 200 μl of lysis buffer (2% Triton X-100, 1% SDS, 100 mM NaCl, 10 mM Tris–HCl (pH 8.0), and 1 mM EDTA (pH 8.0)). The tubes were placed in a dry ice ethanol bath for two minutes until completely frozen, then immersed in a 95 °C water bath for 1 min. Then 100 μl of Tris-saturated phenol (pH 8.0) was added to each tube, vortexed to lyse the cells, then centrifuged for 5 min at 13,000 xg. The aqueous phase of each sample was transferred to a clean 1.5 ml tube, then about 100 μl of chloroform was added to each tube, and centrifuged for 5 min at 13,000 xg at 4 °C. The aqueous layer was then transferred to a new tube containing 400 μl of ice-cold 100% ethanol. The DNA was recovered by centrifugation at 13,000 xg for 5 min, then washed with 70% ethanol. The pellets were resuspended in 1X TE buffer (200 mM Tris–HCl (pH 8.0) and 20 mM EDTA (pH 8.0)) for further use.

#### PCR amplification using ITS

The ITS region of the rDNA was amplified using the primers ITS1 (5'-CGGGATCCGTAGGTGAACCTGCGG-3') and ITS4 (5'-CGGGATCCTCCGCTTATTGATATGC-3'). The final volume of the amplification reaction was 20 ul, which also contained 1 U of Taq polymerase, 20 pmol of each primer, 300 ng of genomic DNA template, 0.2 mM dNTP, and 1.5 mM MgCl2. Using denaturation at 94 °C for 1 min, annealing at 55 °C for 1 min, and extension at 72° C for 2 min, the reactions were conducted for 40 cycles. The amplified DNA fragments were migrated on 1% (w/v) agarose gel electrophoresis for visualisation.

#### ITS DNA sequencing

The PCR products were purified, then sequenced using Applied Biosystems (Sigma, Egypt). The DNA sequences were searched by BLAST (https://blast.ncbi.nlm.nih.gov/Blast.cgi) to identify the closest known sequence related to our query sequences, then deposited into GenBank.

#### Phylogenetic analysis

The evolutionary phylogenetic tree was constructed using the Maximum Likelihood method and Tamura-Nei model [40]. All DNA sequences were aligned by CLUSTALW [41]. Initial tree(s) for the heuristic search were obtained automatically by applying the neighbor- Join and BioNJ algorithms to a matrix of pairwise distances estimated using the Maximum Composite Likelihood (MCL) approach, and then selecting the topology with the superior log likelihood value. The tree is drawn to scale, with branch lengths measured in the number of substitutions per site (above the branches). Evolutionary analyses were conducted in MEGA X [42].

### Antifungal screening of *P. kudriavzevii* strains for plant pathogen fungi

The antagonism of Pk*_*EgyACGEB_O1 and Pk*_*EgyACGEB_O2 strains to the pathogenic plant fungi, *A. terreus, B. cinerea, Bipolaris sp., C. spicifer, R. solani, F. solani, F. circinatum,* and *Fusarium spp*., was assessed. In three replicates, each activated *pichia* strain was streaked to cover the top half of PDA medium agar plates that contain 200 g of potato extract, 20 g of dextrose, 2 g of yeast extract, and 20 g/l of agar, while the other half of the plate was left un-streaked as a control. Two disks were immersed in the activated fungal pathogen and subsequently positioned at the center of both the *Pichia*-streaked and -unstreaked halves of the plate. After inoculation, the plates were placed in a dark incubator and kept at a temperature range of 25–28 °C for 7–14 days, during which each fungus displayed clear growth.

To evaluate the effectiveness of EgyACGEB_O1 and EgyACGEB_O2 filtrates, the five-day growth of each strain in 100 ml of YMPG broth medium was filtrated through a syringe filter with a pore size of 0.22 μm, then mixed with 400 ml of PDA agar medium. The mixture was thoroughly mixed and poured into sterilised Petri dishes. In three plate replicates, one disk from each fungal pathogen was placed at the center of each plate, then incubated at 25 °C for 10–14 days, while fungal growth formation was monitored from day 3 through day 14. Additionally, the same experiment was conducted, but with the *Pichia* YMPG broth filtrates boiled for 5 min before mixing them with the PDA medium. During the culture filtrate assay, we encountered technical difficulties that resulted in the loss of two fungal isolates, *Fusarium circinatum* and *Fusarium spp*. Despite our efforts, we were unable to retrieve these isolates from the source.

### In vivo verification of the *P. kudriavzevii* strains as biocontrol agents

Golden delicious apple fruits free from any signs of damage, wounds, or rot were selected based on uniformity of size. The apples were disinfected with 2% (v/v) sodium hypochlorite for 2 min. After disinfection, the fruits were rinsed with tap water and allowed to air-dry before being utilised in post-harvest biocontrol assays. Two wounds were made on the equator of each fruit with each wound measuring 4 mm in depth and 3 mm in width. To evaluate the efficacy of Pk*_*EgyACGEB_O1 and Pk*_*EgyACGEB_O2 strains as biocontrol agents against *B. cinerea*. The wounds were inoculated with a suspension of either Pk*_*EgyACGEB_O1 or Pk*_*EgyACGEB_O2 at concentrations of 5 × 10^7^ CFUs/ml obtained from 0.5% agar cultures grown for three days. The first treatment received a 25 µl suspension of each *Pichia* strain, while the second treatment received a 50 µl suspension. To ensure the accuracy and reliability of the results, appropriate controls were included in the experimental design. Specifically, a positive control was established using the same concentration of activated commercial yeast at concentrations of 5 × 10^7^ CFUs/ml. On the other hand, the mock treatment was set up using water instead of the biocontrol agent or yeast. After treatment, the fruits were allowed to air-dry for two hours under aseptic conditions before a 25 µl spore suspension of *B. cinerea* at a concentration of 1 × 10^5^ spores/ml was added to each wound. The treated fruits were then placed in covered plastic food trays and stored at a temperature of 25 °C with a relative humidity of about 90% in a growth chamber with a temperature and humidity control system. Disease incidence and wound diameter of apple fruits were determined after 21 days. Each treatment contained four fruits with two replicates per *Pichia* strain, and the experiment was repeated three times.

### Prediction of candidate-secreted proteins of *P. kudriavzevii*

#### Data collection

To predict candidate-secreted proteins of *P. kudriavzevii*, we utilised the genomes of three various strains, CBS573, SD108, and SD129, for further analyses. These strains were selected based on their availability in GenBank. The total protein sequences of CBS573, SD108, and SD129 were collected from the file transfer protocol (FTP) site of the genome deposit of GenBank (https://ftp.ncbi.nlm.nih.gov/genomes/all/GCA/003/054/445/GCA_003054445.1_ASM305444v1/, https://ftp.ncbi.nlm.nih.gov/genomes/all/GCA/000/764/455/GCA_000764455.1_ASM76445v1/, and https://ftp.ncbi.nlm.nih.gov/genomes/all/GCA/001/983/325/GCA_001983325.1_ASM198332v1/), respectively.

#### Effector prediction pipeline

To predict candidate-secreted proteins of *P. kudriavzevii,* total proteins were filtrated using a suite of bioinformatics-based prediction tools. Firstly, the presence of signal peptides was predicted using SignalP, version 5, (https://services.healthtech.dtu.dk/services/SignalP-5.0/, ([43]). The protein sequences with signal peptides resulting from the above step were subsequently submitted for transmembrane domain prediction (https://services.healthtech.dtu.dk/services/TMHMM-2.0/, ([44]). The proteins with zero transmembrane structures (PredHel) were selected for further analysis. We validated the presence of signal peptides and transmembrane domains of all filtrated proteins using Phobius (https://phobius.sbc.su.se/, ([45]).

#### Function annotation

The Blast2GO suite was used to perform the functional annotation of the predicted secreted protein sequences by comparing them to the protein database of the NCBI non-redundant protein database using the BLASTP algorithm. Gene Ontology (GO) terms were then assigned [46].

### Supplementary Information

**Additional file 1: Fig. S1.** Agarose gel electrophoresis of PCR products showing the amplification of a ~600 bp fragment from both EgyACGEB_01 and EgyACGEB_02 isolates.

## Data Availability

GenBank Accession: MZ507552.1 and MZ507554.1.
