# Correction: Towards unlocking the biocontrol potential of *Pichia kudriavzevii* for plant fungal diseases: in vitro and in vivo assessments with candidate secreted protein prediction

**DOI:** 10.1186/s12866-023-03145-9

**Published:** 2023-12-18

**Authors:** Bassma Mahmoud Elkhairy, Nabil Mohamed Salama, Abdalrahman Mohammad Desouki, Ashraf Bakry Abdelrazek, Khaled Abdelaziz Soliman, Samir Abdelaziz Ibrahim, Hala Badr Khalil

**Affiliations:** 1 Department of Genetics, Faculty of Agriculture, Ain Shams University, 68 Hadayek Shoubra, Cairo, 11241 Egypt; 2 Biotechnology Labs, NanoFab Technology Company, 6th October, Giza, Egypt; 3 Department of Plant Pathology, Faculty of Agriculture, Ain Shams University, Postal Code, 68 Hadayek Shoubra, Cairo, 11241 Egypt; 4 Biological Sciences Department, College of Science, King Faisal University, Hofuf, Kingdom of Saudi Arabia

**Correction:** ***BMC Microbiol*** **23, 356 (2023)**

10.1186/s12866-023-03047-w

Following publication of the original article [1], the author would like to add the statement: “The authors express their gratitude to the Deanship of Scientific Research; Vice Presidency for Graduate Studies and Scientific Research; King Faisal University; Al-Ahsa; Saudi Arabia; Grant No. 5102” to the Acknowledgements section. Additionally, the author would like to update the corresponding email address to his current one, which is hkhalil@kfu.edu.sa.

The original article [1] has been corrected.
