# Supplementary figures and images for: Towards unlocking the biocontrol potential of Pichia kudriavzevii for plant fungal diseases: in vitro and in vivo assessments with candidate secreted protein prediction
Source: BMC Microbiol. 2023 Nov 18;23:356. doi: 10.1186/s12866-023-03047-w (PMC10657120; doi:10.1186/s12866-023-03047-w)

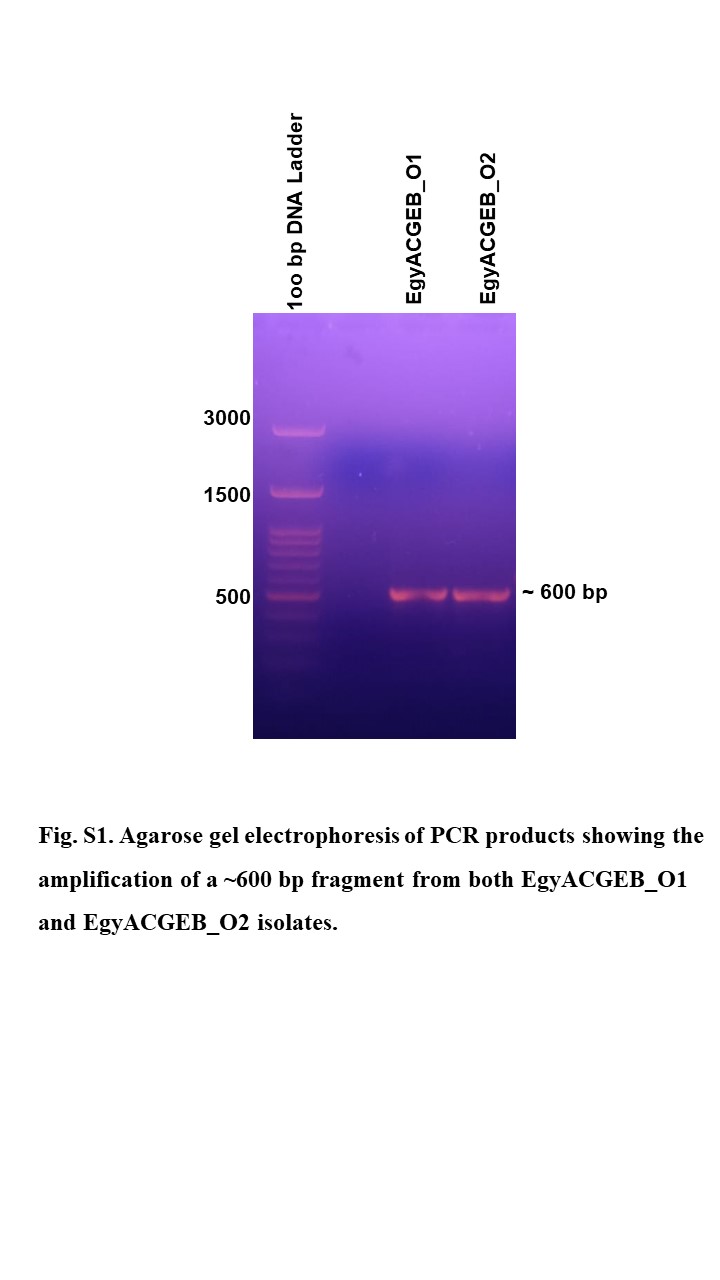

Supplement: Supplementary file 1 — Additional file 1: Fig. S1. Agarose gel electrophoresis of PCR products showing the amplification of a ~600 bp fragment from both EgyACGEB_01 and EgyACGEB_02 isolates. [file 12866_2023_3047_MOESM1_ESM.jpg]
